# The Asylum Workers' Association

**Published:** 1912-05-25

**Authors:** 


### The Asylum Workers' Association.

PRESENTATION OF MEDALS.

The annual general meeting of the Asylum Workers' Association was held on Wednesday afternoon.

Sir W. J. Collins, who presided over the first part of the proceedings, in moving the adoption of the annual report and balance sheet, congratulated all concerned upon an increase in membership, in revenue, and in the balance in hand, and especially upon the increased strength of the Irish Division, which seemed, he said, to be anticipating something in the nature of a Home Rule movement on its own account. Sir William said that he could scarcely believe that it was five years since he first occupied the presidency. He looked back upon nothing during his tenure of office with more pride than the way in which the Association had stood together and achieved the passage of the Superannuation Act of 1909, which for the first time secured the principle of assured and definite pensions for all workers in rate-supported mental hospitals. Whatever modifications of that measure might eventuate, the great triumph of the Association was that it had been able to put it on the statute-book. Certain modifications were needed, however, particularly with regard to the pension age in the case of women, and although the points at issue, if they were much extended, would mean also an extension of the area of possible opposition, yet in the hands of Sir Charles Nicholson he trusted that an amending Bill would go through. He hoped that the legislation on behalf of mental hospital workers would approximate more closely to the Bill which the Association introduced in 1909. It was essential, Sir William thought, that in the present state of affairs their president should be a member of Parliament. He himself was not a member of the Legislature, owing to circumstances over which he had no control, and therefore it was with especial pleasure that he found Sir John Jardine willing to occupy the presidential position. Finally he referred in terms of deep regret to the retirement of Dr. G. E. Shuttleworth from the position of honorary secretary. He had known many a combination of hard head and hard heart, and of soft head and hard heart, but he had never found the hard head and the soft heart so harmoniously blended as in the person of the officer who had served them so well for fifteen years.

The Bishop of Barking seconded the adoption of the report. He said that he had frequent opportunities of coming into touch with the officers of six large mental hospitals in Essex and Hertfordshire, and the necessity was borne in upon him of cultivating some interests, intellectual or otherwise, among asylum workers. The encouragement of a sense of humour among a class of people who were from the nature of their occupation likely to be melancholy seemed most desirable.—Sir William Dunn followed with a eulogy of Sir William Collins, and the report and balance sheet were then adopted.

A special vote of thanks to the retiring President was most warmly accorded on the motion of Dr. E. S. Pasmore (Croydon Mental Hospital), seconded by the Rev. H. Whittaker, M.D., chaplain of Banstead Mental Hospital. In responding, Sir William Collins caused laughter by quoting the sardonic proverb, that wherever you went you found latitude and longitude, but never gratitude. This was not true, at any rate of mental hospital workers, among whom he had found gratitude in abundance.

Dr. T. Drapes, of Enniscorthy Mental Hospital, then formally moved the election of Sir John Jardine, M.P., as President, and this was seconded by Dr. J. Carswell, of Glasgow.

Sir John Jardine, on taking the chair, referred at some length to his experience of mental hospital organisation in India, and to legislative work in the future, particularly to the need of a new Bill to amend the deficiencies in the legislation of 1909. He looked for a bigger demand for the services of capable workers and nurses as a consequence of projected legislation with regard to the feeble-minded.

The list of vice-presidents, officers, and members of the executive committee, with a few alterations from the list of the previous year, was proposed by Dr. W. J. Seward, seconded by Dr. Percy Smith, and carried. The place of honorary secretary was left vacant, and it was announced that Dr. Shuttleworth had consented to continue to act until his successor was appointed. A proposition that the executive committee should be empowered to appoint a secretary, the appointment to come up for confirmation at the next general meeting, was agreed to, after being proposed from the chair, and seconded by the Rev. C. Major Jenkins (Darenth Colony), who strongly urged the necessity of appointing a medical man as honorary secretary.

The proceedings concluded with the interesting ceremony of the presentation of medals. Attendant N. Livingstone, of Argyle and Bute Asylum, and Nurse M. J. Goodchild, of Banstead, received gold medals for forty years and thirty-four years' service respectively. A silver medal was awarded to Attendant A. Walters, of Grahamstown, and five bronze medals to Attendants C. M. Clarke, G. H. Smith, W. J. Burgess, G. Borrill, and W. A. Marshall.

